# TGM2 accelerates migration and differentiation of BMSCs by activating Wnt/β-catenin signaling

**DOI:** 10.1186/s13018-023-03656-1

**Published:** 2023-03-05

**Authors:** Feng Liu, Mingzheng Wu, Xixia Wu, Dan Chen, Ming Xie, Hao Pan

**Affiliations:** Department of Orthopedics, Wuhan Fourth Hospital, Wuhan, China

**Keywords:** Bone defects, Tissue engineering, KLD-12, SDF-1, BMSCs

## Abstract

**Background:**

Transglutaminase 2 (TGM2) is a gene previously reported to be associated with the differentiation of bone marrow mesenchymal stem cells (BMSCs). The study was developed to reveal the impact of TGM2 on the migration and differentiation of BMSCs.

**Methods:**

Cells were isolated from bone marrow of mice and then the surface antigens were identified by flow cytometry. Wound healing assays were conducted to assess the migratory ability of BMSCs. The mRNA levels of TGM2 and osteoblast-associated genes (ALP, OCN, and RUNX2) were subjected to RT-qPCR analysis, and protein levels of these genes as well as β-catenin were quantitated by western blotting. Alizarin red staining was conducted for detection of osteogenic ability. The activation of Wnt signaling was assessed by TOP/FOP flash assays.

**Results:**

Surface antigens were positively identified in MSCs, indicating good multidirectional differentiation ability of cells. TGM2 silencing suppressed BMSC migration while weakening mRNA and protein levels of osteoblast-associated genes. While TGM2 overexpression exerts the opposite impact on cell migration and expression levels of osteoblast-associated genes. Additionally, overexpressed TGM2 promotes the mineralization of BMSCs according to results of Alizarin red staining. Moreover, TGM2 activated the Wnt/β-catenin signaling, and DKK1 (an inhibitor of Wnt signaling) reversed the promoting influence of TGM2 on cell migration and differentiation.

**Conclusion:**

TGM2 promotes the migration and differentiation of BMSCs via activation of the Wnt/β-catenin signaling.

**Supplementary Information:**

The online version contains supplementary material available at 10.1186/s13018-023-03656-1.

## Introduction

Bone marrow mesenchymal stem cell (BMSC) is considered as a cellular substrate for multiple tissue repair due to their presumptive plasticity, immune tolerance and genetic stability [1]. In addition, BMSCs are a population of fibroblast-like and non-hematopoietic cells with characteristics of self-renewal and huge differentiation potential [2, 3].

Transglutaminase 2 (TGM2) has been reported to be a gene associated with BMSC differentiation according to a previous report [4]. TGM2 is involved in bone-related diseases. For example, fluoride can cause bone damage by upregulating TGM2 and activate the PI3K/Akt signaling [5]. As to osteosarcoma, knockdown of TGM2 promotes the chemosensitivity of osteosarcoma cells by inhibiting Akt and MAPK signaling pathways [6]. Additionally, TGM2 promotes metastatic phenotypes of osteosarcoma cells and is responsible for osteosarcoma stem-like properties [7].

Some studies put forward that TGM2 might be involved in the fate regulation of BMSCs via a few signaling pathways including canonical WNT [8, 9]. Han et al. [8] reported that TGM2 is induced in articular cartilage and aggravates the severity of surgically induced osteoarthritis by promoting canonical Wnt signaling via β-catenin stabilization. TMG2 promotes calcification and osteoblastic-transformation in vascular smooth muscle cells by activating the β-catenin signaling pathway [9]. Wnt/β-catenin signaling plays a regulatory role in cell proliferation, migration, and cell to cell adhesion [10]. The signaling was also reported to participate in BMSC differentiation. According to the report from Xiao-Jun Chen et al*.*, the Wnt/β-catenin signaling is stimulated by polydatin via BMP2, and then target genes of the signaling, including β-catenin, cyclin D, Lef1, and c-jun were increased, finally promoting osteogenic differentiation [11]. Moreover, STAT4 overexpression in gastric cancer cells activates the Wnt7a/β-catenin signaling in normal fibroblasts (NFs) and transforms NFs and BMSC to cancer associated fibroblasts [12]. Therefore, we hypothesized that TMG2 may regulate the Wnt/β-catenin signaling in BMSCs.

In this work, we aimed to reveal the role of TGM2 in regulating the differentiation and migration of BMSCs and its association with the Wnt signaling, which may extend our understanding of the functions of TGM2 in bone-related diseases.

## Materials and methods

### Animals

Male C57 black mice (8 weeks, average weight: 20 g) were purchased from the Hubei Provincial Center for Disease Control and Prevention (Wuhan, Hubei, China). All mice were kept under standard conditions individually. All animal experiments were conducted in the ABSL-III laboratory according to the Guidelines of Animal Use and Care.

### Cell culture

MSCs were isolated from mice as previously described [13]. In brief, after the femurs of mice were dissected out and crushed, the contents were digested in Dulbecco's modified Eagle's medium (DMEM; #D0697; Sigma-Aldrich, St. Louis, USA). The resulting suspension was pelleted by 5 min of centrifugation at 1500 × g using a centrifugal machine (Hettich, Germany). The remaining cells were then beaten in the culture medium and transferred to an incubator (Thermo Fisher Scientific, Waltham, USA; conditions: 37 °C, 5% CO_2_ and 95% humidity).

### Cell transfection

The pcDNA3.1 vector (Invitrogen, Carlsbad, USA) containing TGM2 sequence was employed for TGM2 overexpression. Short hairpin RNA targeting TGM2 (sh-TGM2) was used to silence TGM2 expression. These plasmids as well as the corresponding negative control (NC) plasmids, empty pcDNA3.1 vector and sh-NC plasmid, were purchased and synthesized by Genepharma (Shanghai, China) and then were transfected into BMSCs using Lipofectamine 3000 reagent (Life Technologies, Carlsbad, USA) following the manufacture’s recommendations.

### Identification of BMSC surface antigens

After BMSCs digestion with trypsin and centrifugation at 1000 rpm for 3 min, the supernatant was removed. After washing, the cells were supplemented with antibodies (Abcam, Cambridge, USA) against CD90 (#ab3105), CD73 (#ab288154), CD105 (#ab221675), CD34 (#ab23830) and CD45 (#ab10558) in dilution with phosphate-buffered saline (#P4417; Sigma Aldrich, St. Louis, MO, USA) for 30 min of incubation. Afterwards, centrifugation of cell suspension (1000 rpm, 3 min) was performed followed by discarding of the supernatant. A flow cytometry (BD Biosciences, San Jose, USA) was applied for the detection of cell surface antigens.

### Wound healing assay

The assay was conducted after BMSCs were plated into 6-well plates and reached 70–80% confluence. A sterile pipette tip (200 μl) was used to make wounds in the monolayers of cells. After that, fresh growth medium (#D0697; Sigma-Aldrich, St. Louis, USA) was replaced. The degree of wound healing was calculated by taking microscopic images of the scratch at 0 h and 24 h.

### Reverse transcription PCR

RIzol reagent (#15596026; Invitrogen, Carlsbad, USA) was utilized for extracting RNA (50 μg) from the BMSCs. DNase I (#HY-P72974; Medchem Express, Monmouth Junction, USA) treatment was performed to digest RNA samples and then reverse transcription was performed utilizing Superscript II Reverse Transcriptase (#18064014; Invitrogen), and PCR was performed utilizing GoTaq qPCR Master Mix (#A6001; Promega, Madison, USA) following the user’s recommendations. The sequences of primes are listed in Table 1.

**Table 1:** Sequences of primes used for RT-qPCR

Gene	Sequence (5′→3′)
*Mus GAPDH*	
F	ATGGGTGTGAACCACGAGA
R	CAGGGATGATGTTCTGGGCA
*Mus TGM2*	
F	CTTCTACTGGCTACCAGGG
R	AGGTACACATCATCGGCTG
*Mus OCN*	
F	TGAGGACCATCTTTCTGCTCA
R	TGGTCTGATAGCTCGTCACA
*Mus ALP*	
F	TGCCCTGAAACTCCAAAAGC
R	CTTCACGCCACACAAGTAGG
*Mus Runx2*	
F	AGATGGGACTGTGGTTACCG
R	TAGCTCTGTGGTAAGTGGCC

*F*, forward; *R*, reverse

### Western blotting

rotein contents were extracted from cells using radio immunoprecipitation lysis buffer (#89901; Thermo Fisher Scientific, Waltham, USA) containing proteinase and phosphatase inhibitors (Thermo Fisher Scientific), and the concentration was determined using a BCA protein assay kit (#P0011; Beyotime, Shanghai, China). After separated by SDS-PAGE, protein samples were transferred to the PVDF membrane (#GVWP02500; Sigma-Aldrich, St. Louis, USA). GAPDH was used as a loading reference. The membrane was subsequently incubated with primary antibodies against TGM2 (#ab2386; 1:1000, Abcam, Cambridge, UK), alkaline phosphatase (ALP; #ab229126; 1:500, Abcam), osteocalcin (OCN; #ab93876; 1:1000, Abcam), and runt-related transcription factor 2 (RUNX2; #ab236639; 1:1000, Abcam) at 4 °C overnight followed by further incubation with secondary antibodies. An enhanced chemiluminescence system (Kodak, Rochester, USA) was applied to visualize the signals on the membrane. ImageJ software (NIH, Bethesda, USA) was used analyze the signal intensity of the blots.

### Alizarin red staining for mineralization assessment

After 3 weeks of appropriate BMSC culture, cells were stained with alizarin red (#60504ES25; Yeasen, Shanghai, China). First, the cells were immobilized for 30 min using 4% neutral formaldehyde solution (#BWZ6716; Beijing, China). Then, the remaining liquid was discarded. Cells were then stained for 5 min with an Alizarin Red solution (pH = 4.2, #RASMX-90021, Cyagen Biosciences, Guangzhou, China). Finally, the Alizarin Red solution was discarded. After air-dried, the mineralized nodes were investigated by an inverted microscope (Olympus, Tokyo, Japan).

### TOP/FOP flash assay

BMSCs were plated into 24-well plates (2 × 10^4^/well) for 12 h. Then, Topflash or FOPflash luciferase reporters (#D2506; Beyotime, Haimen, China) were transfected into cells according to the guide. Forty-eight hours after transfection, luciferase activity was detected using the Luciferase Assay System (Promega, Madison, USA).

### Statistical analysis

Data were analyzed using the SPSS 17.0 software package (SPSS Inc., Chicago, USA) and were shown as the mean ± SD. One-way analysis of variance (ANOVA) and unpaired student’s *t* tests were used for difference evaluation between groups. Two-tailed *p* value less than 0.05 was deemed as of statistical significance.

## Results

### Identification of BMSCs

**Fig. 1:**
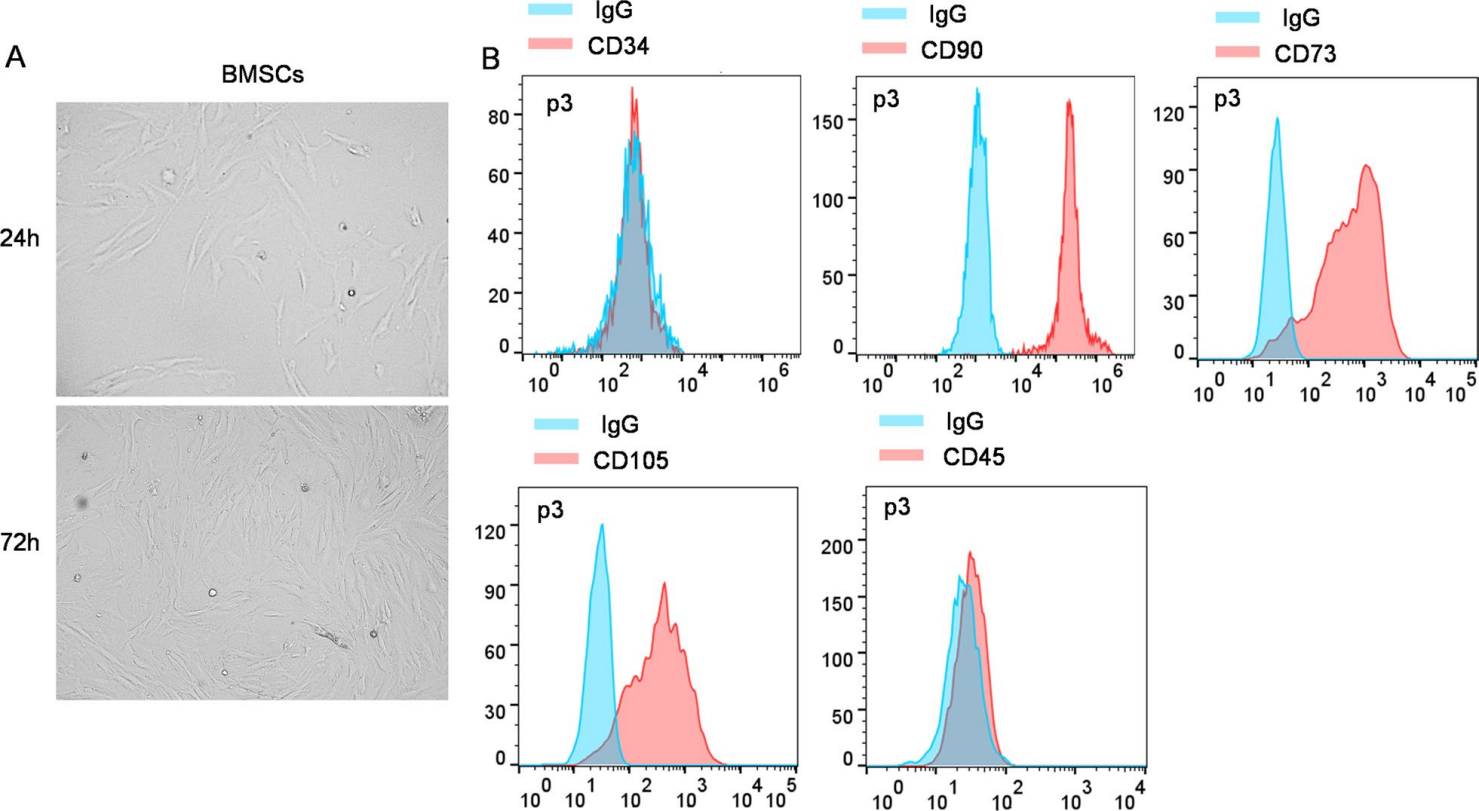
Identification of BMSCs. **A** After 24 h or 72 h of cell culture, the morphology of BMSCs was observed. The cells were in a long spindle shape. **B** Expression levels of surface antigens CD34(−), CD90(+), CD105(+), CD45(−) were analyzed using flow cytometry

After cell culture for 1 day or 3 days, cell morphology was observed. BMSCs were characterized with sharp boundary and good refraction, and some adhered cells were distributed as a vortex (Fig. 1A). In addition, flow cytometry analysis was performed to measure expression levels of cell surface antigens, positive-antigens CD90, CD73 and CD105 as well as negative-antigens CD34 and CD45. As shown by Fig. 1B, the decrease in CD34(−) and CD45(−) levels and the increase in CD90(+), CD73(+), and CD105(+) expression suggested the high purity of the extracted BMSCs.

### Silencing of TGM2 inhibits cell migration and TGM2 overexpression promotes cell migration

**Fig. 2:**
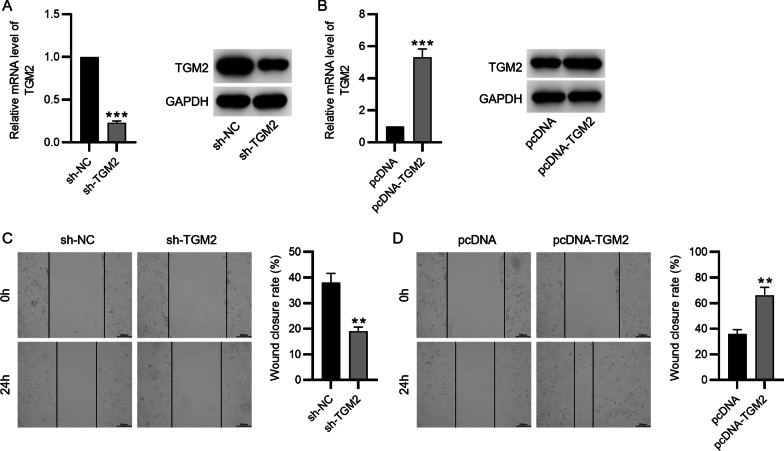
Silencing of TGM2 inhibits cell migration while TGM2 overexpression promotes cell migration. **A** and **B** Knockdown efficacy and overexpressing efficacy of sh-TGM2 and pcDNA-TGM2 after transfection were evaluated by PCR and western blotting. **C** and **D** The migration of BMSCs silencing or overexpressing TGM2 was detected by wound healing assays. **p < 0.01, ***p < 0.001

To quantify the migratory capability of BMSCs after indicated treatment, we performed a scratch wound healing assay at 0 h and 24 h. First, the knockdown and overexpression efficacies were detected by qPCR and western blot analysis. The decrease in TGM2 mRNA and protein levels after sh-RGM2 transfection (Fig. 2A) and the elevation of TGM2 levels after pcDNA-TGM2 transfection (Fig. 2B) were observed. For the wound healing assay, the migratory capability of BMSCs was significantly inhibited in sh-TGM2 group (Fig. 2C) while being promoted in BMSCs overexpressing TGM2 (Fig. 2D). These results suggested that TGM2 promotes BMSC migration.

### TGM2 increases ALP, OCN, and Runx2 expression in BMSCs

**Fig. 3:**
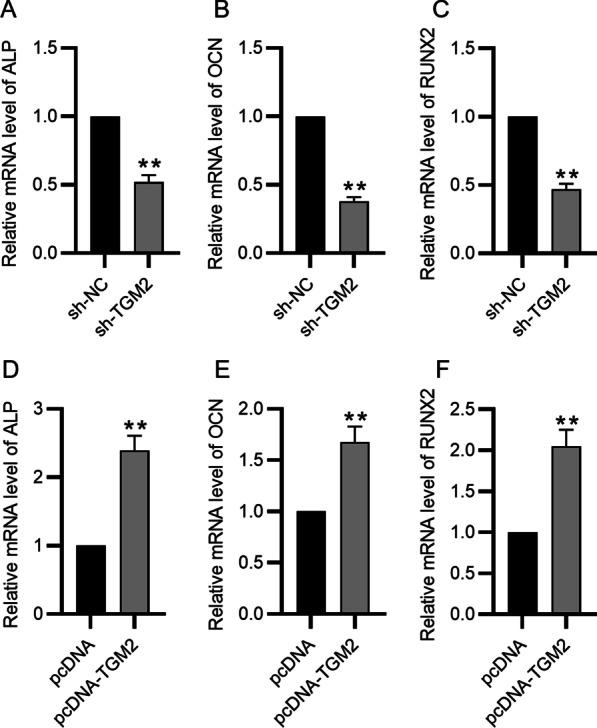
TGM2 increases ALP, OCN, and Runx2 mRNA expression in BMSCs. **A**–**C** RT-qPCR analysis of ALP, OCN, and Runx2 expression in BMSCs with sh-TGM2/sh-NC. **D**–**F** The impact of TGM2 overexpression on ALP, OCN, and Runx2 mRNA expression in BMSCs was quantified by qPCR analysis. **p < 0.01

ALP, OCN, and Runx2 are common bone differentiation markers of osteogenic differentiation [14]. In this work, ALP, OCN, and Runx2 expression in sh-TGM2 group was greatly weakened compared with that in the control group (Fig. 3A–C). TGM2 overexpression exerts the opposite effect on mRNA levels of these genes. Increased ALP, OCN and Runx2 levels were detected in pcDNA-TGM2 group (Fig. 3D–F).

**Fig. 4:**
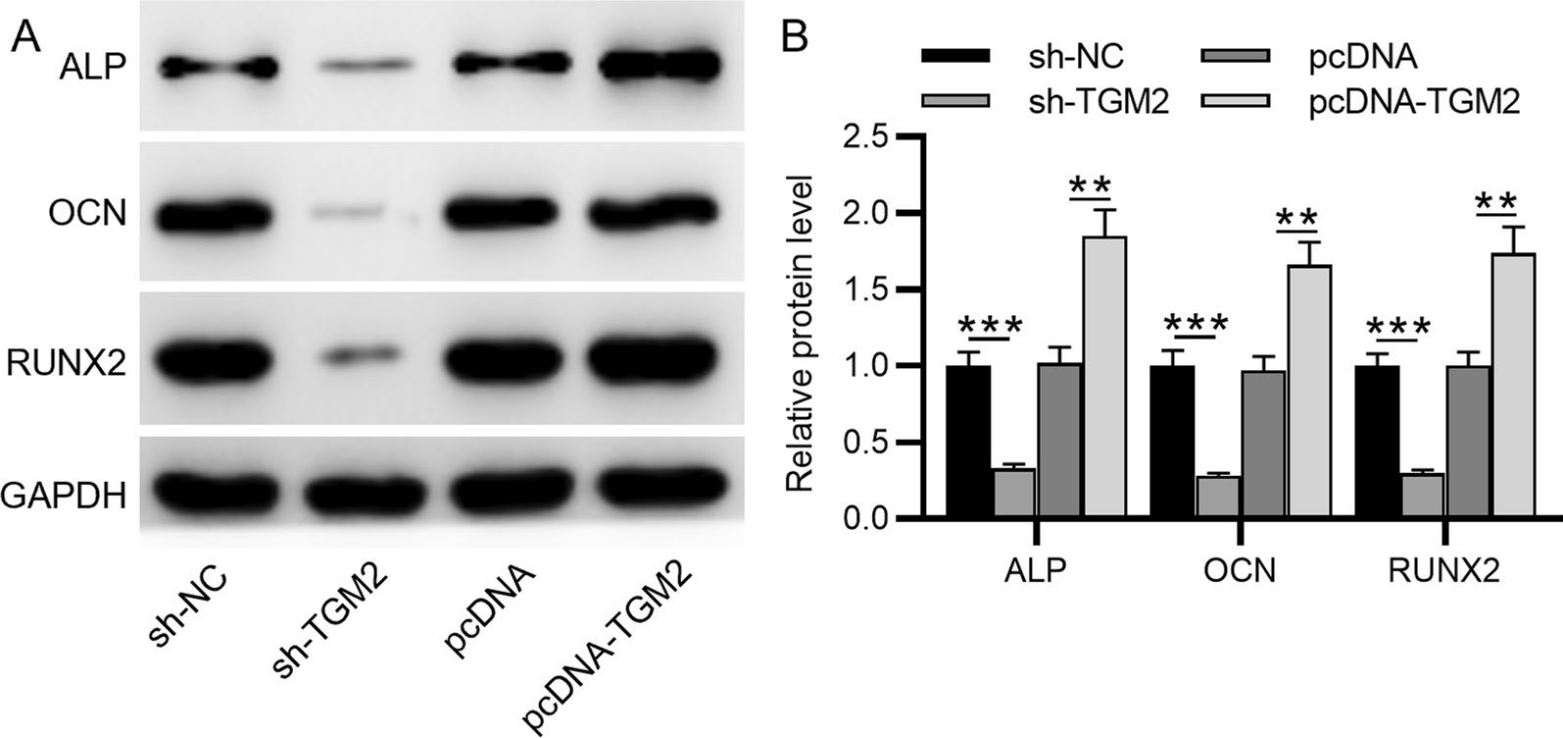
The impact of TGM2 on ALP, OCN, and RUNX2 protein levels. **A** ALP, OCN, and RUNX2 protein levels in response to TGM2 deficiency or overexpression were measured by western blotting. **B** The quantification of signal intensity of the blots. **p < 0.01, ***p < 0.001

Moreover, ALP, OCN and Runx2 protein expression was also inhibited by TGM2 depletion and enhanced by overexpressed TGM2 (Fig. 4A–B). This finding of our study demonstrated that TGM2 could increase the secretion of ALP, OCN, Runx2, indicating the differentiation of BMSCs into osteoblasts.

### TGM2 promotes the mineralization of BMSCs

**Fig. 5:**
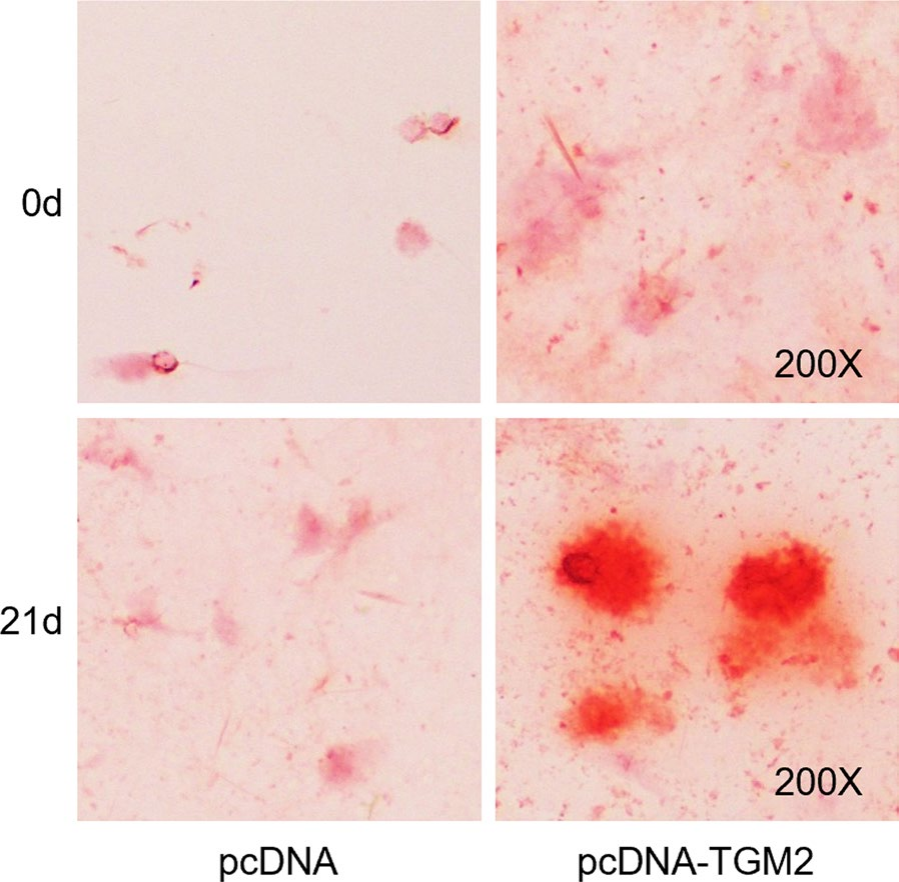
TGM2 promotes the mineralization of BMSCs. Images for Alizarin red staining in control group and TGM2 overexpression group at day 0 and day 21. The uncropped images were provided as Additional files 1, 2, 3 and 4: Figures.

The formation of calcium nodules can reflect the degree of BMSC differentiation into osteoblasts [15]. After 3 weeks, the pcDNA-TGM2 group had stronger osteogenesis induction ability, as evidenced by the increase in the number of calcium nodules as well as the deeply stained cells (Fig. 5). Original images for Alizarin red staining are provided as Additional files 1, 2, 3 and 4: Figures. Therefore, the results indicated that TGM2 overexpression promotes the mineralization degree of BMSCs and might promote osteogenic differentiation.

### TGM2 activates the Wnt/β-catenin signaling to promote cell migration and differentiation

**Fig. 6:**
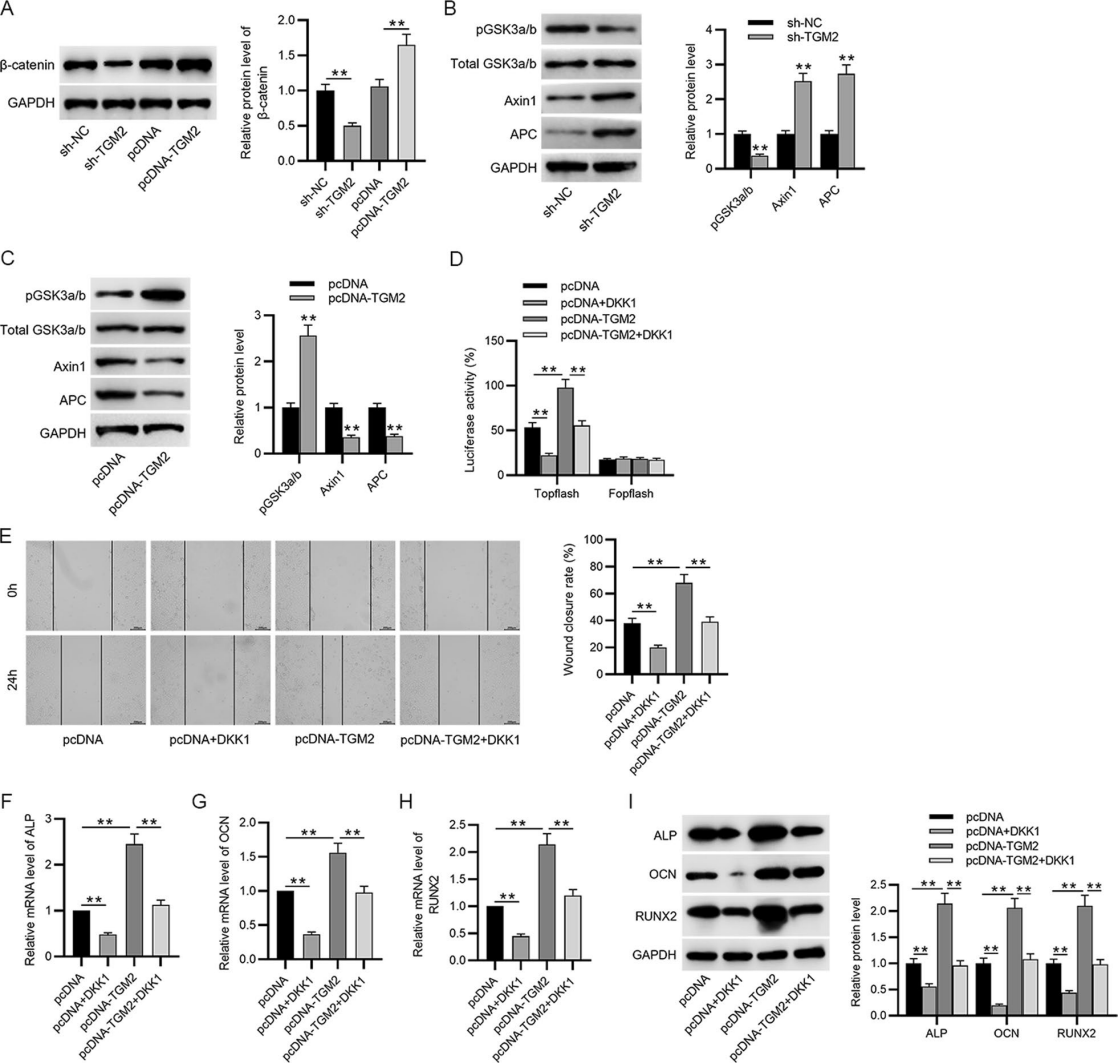
TGM2 activates the Wnt/β-catenin signaling to promote cell migration and differentiation. **A** β-catenin protein level in BMSCs silencing or overexpressing TGM2 was quantitated by western blotting. **B** and **C** Phosphorylated GSK3a/b, Axin1, and APC protein levels in response to TGM2 deficiency or overexpression were quantified using western blotting. **D** Topflash activities of four groups (pcDNA, pcDNA + DKK1, pcDNA-TGM2, and pcDNA-TGM2 + DKK1) were measured by the luciferase activity assay. **E** Wound healing assay was performed to detect cell migration in the above-mentioned four groups. **F**–**I** ALP, OCN, and RUNX2 mRNA and protein levels in BMSCs of the above four groups were detected using qPCR and western blotting. **p < 0.01

Canonical Wnt/β-catenin is closely associated with osteogenesis according to previous reports [16]. The Wnt proteins are essential for skeletal formation and are implicated with chondrogenesis formation, the differentiation and synthesis of bone matrix by osteoblasts [17, 18]. In addition, TGM2 has been reported to activate Wnt/β-catenin in osteoarthritis and vascular smooth muscle cells [8, 9]. Hence, we wondered whether TGM2 can activate the Wnt/β-catenin in BMSCs and weather TGM2 affects BMSC migration and differentiation by controlling the activation of Wnt signaling. Dickkopf Wnt signaling pathway inhibitor 1 (DKK1) was used to silence the Wnt signaling in BMSCs. Western blotting revealed that β-catenin protein level was silenced by TGM2 depletion and promoted by TGM2 overexpression compared to the control group (Fig. 6A). In addition, some proteins associated with β-catenin destruction complex (phosphorylated GSK3a/b, total GSK3a/b, Axin1, and APC) in response to TGM2 alteration were quantified using western blotting. The results revealed that phosphorylated level of GSK3a/b was decreased after TGM2 depletion while Axin1 and APC levels were increased under the same treatment (Fig. 6B), and TGM2 overexpression exerted the opposite influence on phosphorylated GSK3a/b, Axin1, and APC protein levels (Fig. 6C). Moreover, Topflash activity was lowered after DKK1 treatment and was greatly increased by TGM2 overexpression (Fig. 6D). Additionally, combination of DKK1 and TGM2 treatment countervailed TGM2-induced increase in Topflash activity (Fig. 6D). The results indicated that TGM2 can activate the Wnt/β-catenin signaling in BMSCs. According to wound healing assays, the wound closure rate was reduced by DKK1 treatment, and TGM2 overexpression promoted cell migration (Fig. 6E). Importantly, the promotive impact on cell migration induced by TGM2 overexpression was reversed by DKK1 treatment (Fig. 6E). In addition, DKK1 treatment also reversed the increase in ALP, OCN, and RUNX2 mRNA and protein levels induced by TGM2 overexpression (Fig. 6F–I). Overall, it can be concluded that silencing of Wnt/β-catenin reverses the promotive impact of TGM2 on BMSC migration and differentiation, indicating that TGM2 promotes BMSC migration and differentiation by inducing the activation of Wnt/β-catenin signaling.

## Discussion

The main findings of this work can be concluded as follow (1) TGM2 enhances the migration and repair capabilities of mouse BMSCs; (2) TGM2 elevates expression levels of ALP, OCN, Runx2 and promoted the biological activity of BMSCs; (3) TGM2 plays a significant role in BMSCs conversion to osteoblasts in vitro.

TGM2 is distributed in bone and cartilage and can induce the hypertrophic differentiation of joint chondrocytes and osteoarthritis formation [19, 20]. It is reported that TGM2 promotes the expression of osteogenic genes (OC, OPN, and Runx2) and enhances ALP activity [20], and TGM silencing reduces OC and collagen-I expression as well as ALP activity [21]. Consistently, TGM2 in this study was found to promote mRNA and protein levels of osteoblast-related genes (ALP, OCN, and RUNX2) in BMSCs. In addition, TGM2 has been revealed to participate in the initiation and modulation of the mineralization process in osteosarcoma cell line SAOS-2 [21]. Similarly, in this study, TGM2 increased the number of calcium nodules and promoted the mineralization degree of BMSCs. Despite BMSCs, ilium MSCs also displays high expression of TGM2 compared to jaw MSCs, showing chondrogenic differentiation potential of TGM2 [22]. It can be seen that TGM2 has huge potential in skeletal regeneration, and more experiments deserve to be conducted for further exploration of TGM2 for regenerative therapy.

Previously, TGM2 was discovered to activate focal adhesion kinase and then lead to the activation of anti-apoptotic signaling pathways such as PI3K/AKT, thereby accelerating cell proliferation [5, 23]. In addition, TGM2 can influence cell to cell interactions by regulating the formation of cellular matrices through secreting extracellular regulation factors [24]. The Wnt/β-catenin is also a signaling related to cell proliferation, migration, and cell–cell interactions [10]. Moreover, the Wnt signaling is widely reported to be associated with BMSC osteogenesis. For instance, adiponectin contributes to BMSC differentiation and bone formation via the Wnt signaling [25]. miR-486-3p targets downstream gene CTNNBIPI and thus activates the Wnt/β-catenin signaling, thereby promoting osteogenic differentiation [26]. In this work, TGM2 was reported to activate the Wnt/β-catenin signaling, which was consistent with previous studies revealing the relation of TGM2 and Wnt signaling in osteoarthritis and vascular calcification as well as other diseases such as colorectal cancer and bronchopulmonary dysplasia [8, 9, 27–29].

In conclusion, TGM2 enhances BMSC wound healing ability, elevates RNA and protein levels of osteoblast-associated genes while promoting the mineralization degree of BMSCs. Additionally, TGM2 depletion exerts the opposite effect on BMSC migration and differentiation. Moreover, TGM2 activates the Wnt signaling, and inhibition of the signaling reverses the promoting influence on cell migration and differentiation caused by TGM2 overexpression. Therefore, TGM2 promotes the differentiation and migration of BMSCs through activation of the Wnt/β-catenin signaling. The study enriches the understanding of TGM2/Wnt/β-catenin pathway in BMSCs, which may be an effective strategy for bone-associated diseases such as osteoarthritis, spinal cord injury, and cartilage injury.

## Supplementary Information

**Additional file 1**: Uncropped images for Alizarin red staining

**Additional file 2**: Uncropped images for Alizarin red staining

**Additional file 3**: Uncropped images for Alizarin red staining

**Additional file 4**: Uncropped images for Alizarin red staining
