# Supplementary figures and images for: TGM2 accelerates migration and differentiation of BMSCs by activating Wnt/β-catenin signaling
Source: J Orthop Surg Res. 2023 Mar 5;18:168. doi: 10.1186/s13018-023-03656-1 (PMC9985845; doi:10.1186/s13018-023-03656-1)

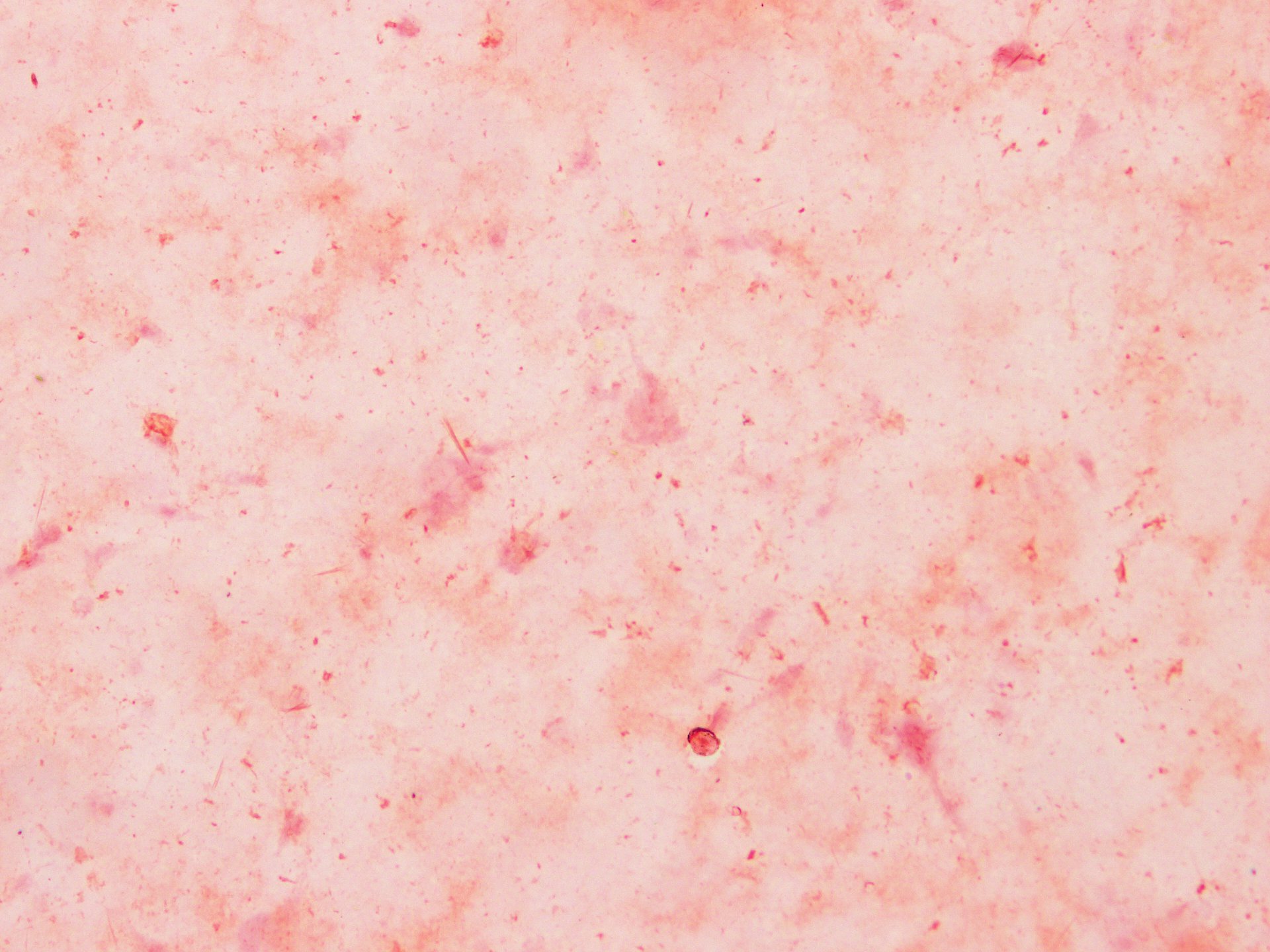

Supplement: Supplementary file 1 — Additional file 1: Uncropped images for Alizarin red staining [file 13018_2023_3656_MOESM1_ESM.jpg]

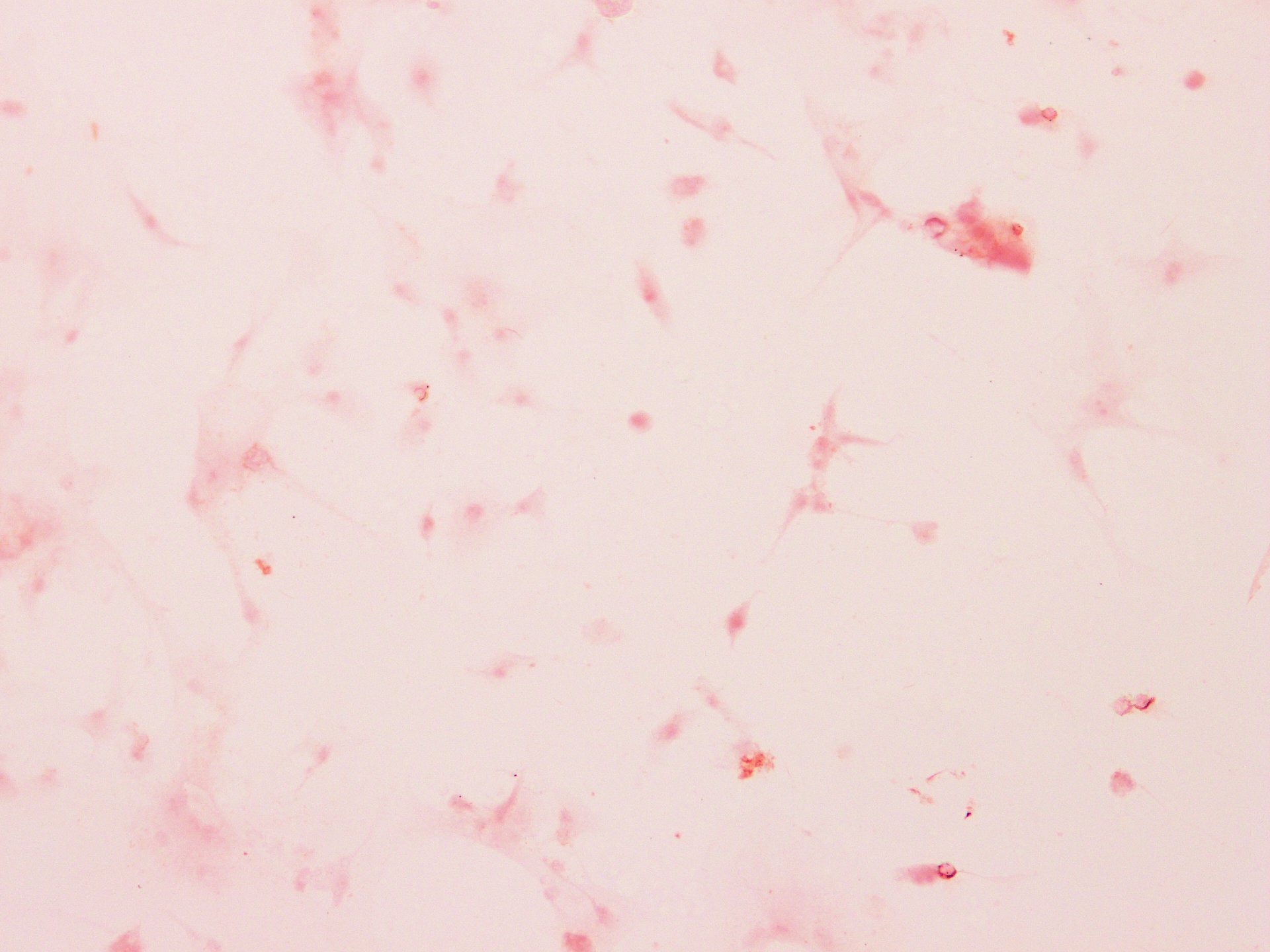

Supplement: Supplementary file 2 — Additional file 2: Uncropped images for Alizarin red staining [file 13018_2023_3656_MOESM2_ESM.jpg]

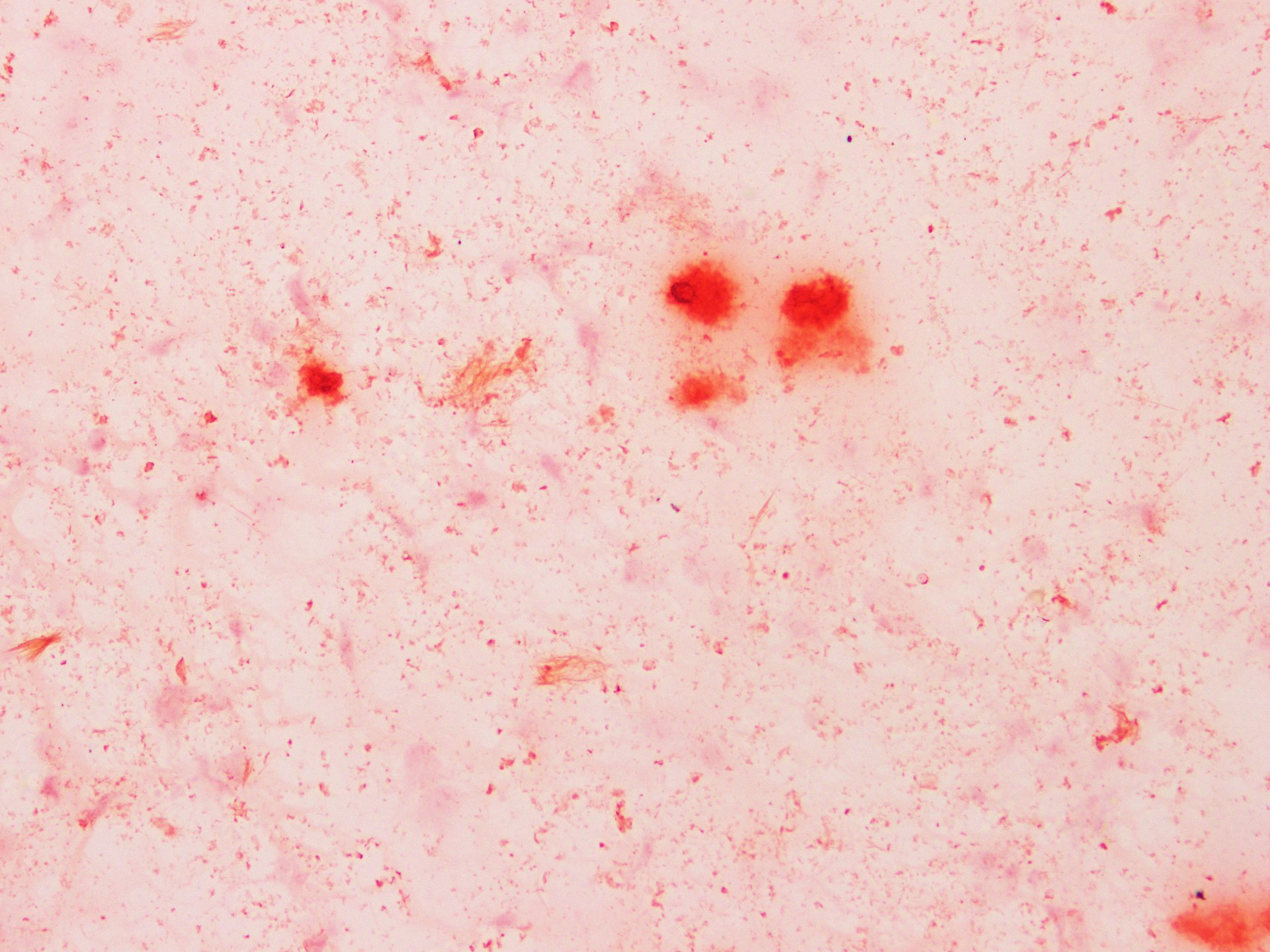

Supplement: Supplementary file 3 — Additional file 3: Uncropped images for Alizarin red staining [file 13018_2023_3656_MOESM3_ESM.jpg]

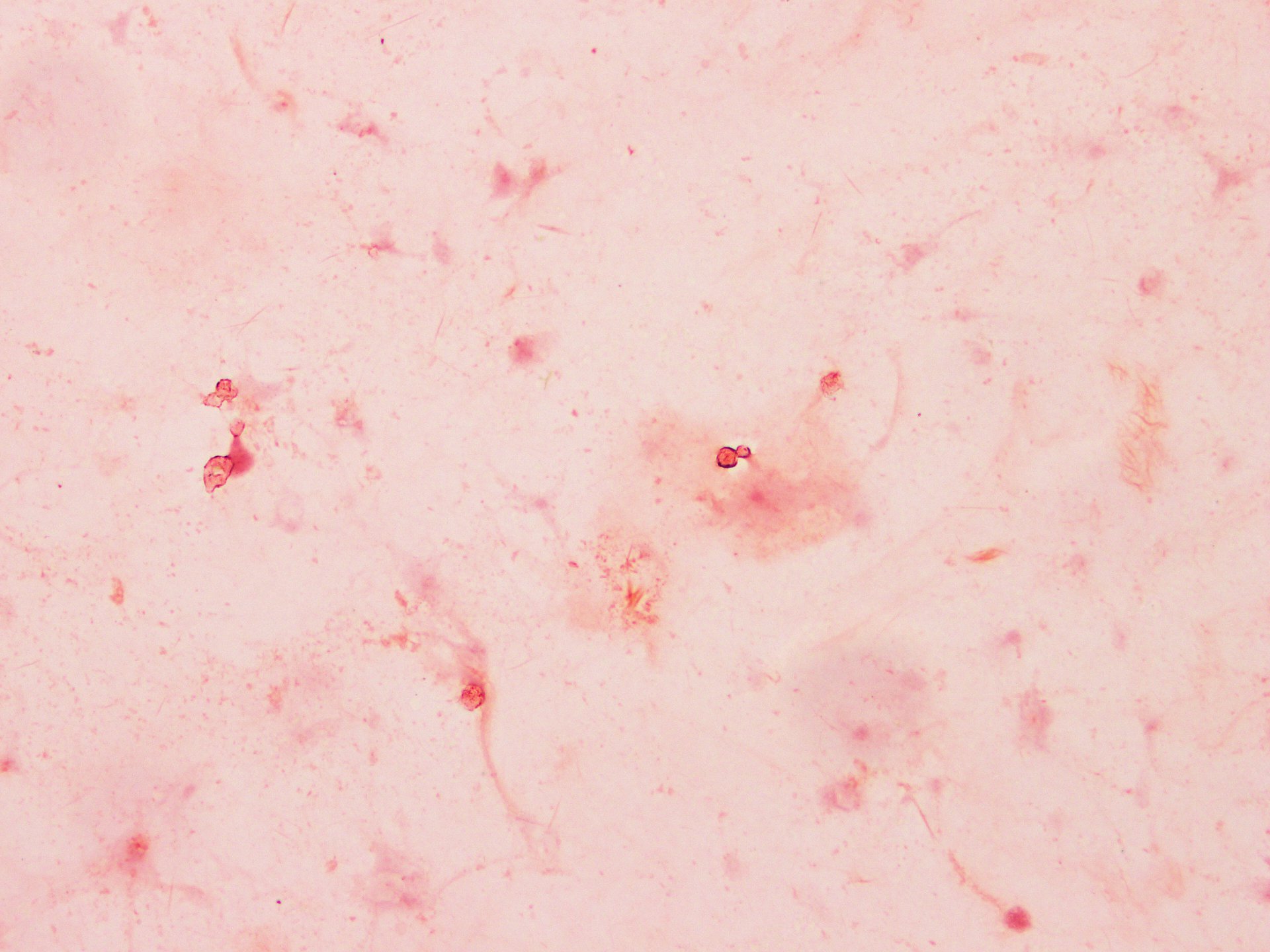

Supplement: Supplementary file 4 — Additional file 4: Uncropped images for Alizarin red staining [file 13018_2023_3656_MOESM4_ESM.jpg]
